# Supplementary figures and images for: Reversible acetylation of HDAC8 regulates cell cycle
Source: EMBO Rep. 2024 Jul 23;25(9):13. doi: 10.1038/s44319-024-00210-w (PMC11387496; doi:10.1038/s44319-024-00210-w)

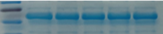

Supplement: Supplementary file 2 — Source data Fig. 1 [file 44319_2024_210_MOESM2_ESM.zip › Figure 1/1C/Coomassie blue stain.tif]

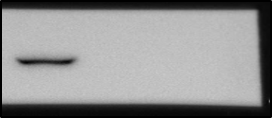

Supplement: Supplementary file 2 — Source data Fig. 1 [file 44319_2024_210_MOESM2_ESM.zip › Figure 1/1E/western AcK202.tif]

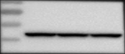

Supplement: Supplementary file 2 — Source data Fig. 1 [file 44319_2024_210_MOESM2_ESM.zip › Figure 1/1E/western FLAG.tif]

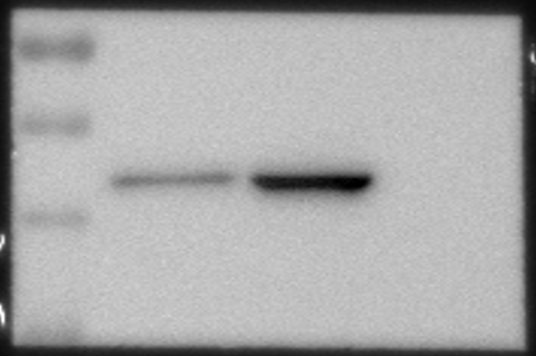

Supplement: Supplementary file 2 — Source data Fig. 1 [file 44319_2024_210_MOESM2_ESM.zip › Figure 1/1F/western AcK.tif]

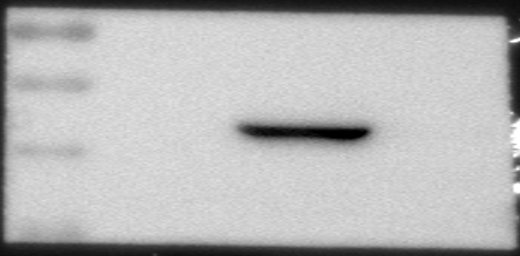

Supplement: Supplementary file 2 — Source data Fig. 1 [file 44319_2024_210_MOESM2_ESM.zip › Figure 1/1F/western AcK202.tif]

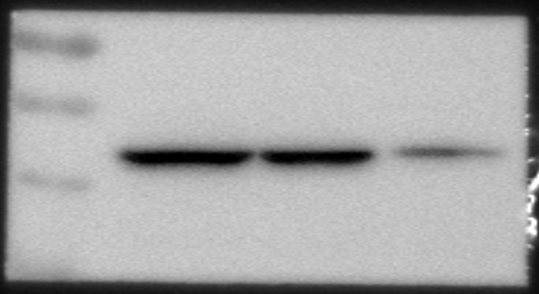

Supplement: Supplementary file 2 — Source data Fig. 1 [file 44319_2024_210_MOESM2_ESM.zip › Figure 1/1F/western HDAC8.tif]

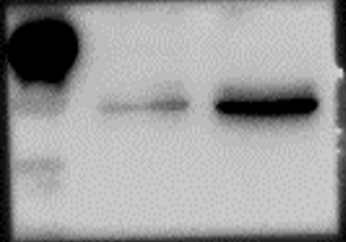

Supplement: Supplementary file 2 — Source data Fig. 1 [file 44319_2024_210_MOESM2_ESM.zip › Figure 1/1G/western AcK.tif]

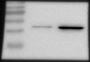

Supplement: Supplementary file 2 — Source data Fig. 1 [file 44319_2024_210_MOESM2_ESM.zip › Figure 1/1G/western AcK202.tif]

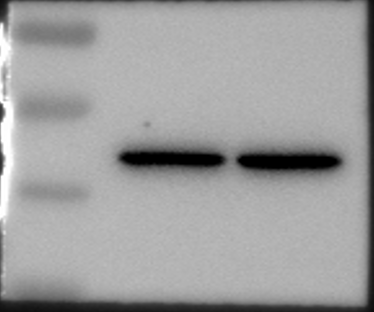

Supplement: Supplementary file 2 — Source data Fig. 1 [file 44319_2024_210_MOESM2_ESM.zip › Figure 1/1G/western FLAG INPUT.tif]

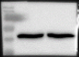

Supplement: Supplementary file 2 — Source data Fig. 1 [file 44319_2024_210_MOESM2_ESM.zip › Figure 1/1G/western FLAG.tif]

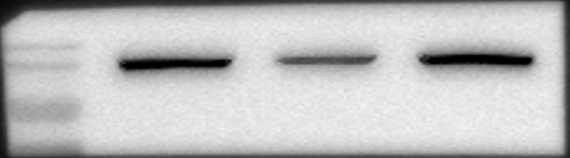

Supplement: Supplementary file 2 — Source data Fig. 1 [file 44319_2024_210_MOESM2_ESM.zip › Figure 1/1H/western AcSMC3.tif]

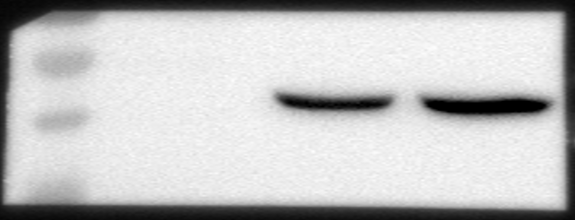

Supplement: Supplementary file 2 — Source data Fig. 1 [file 44319_2024_210_MOESM2_ESM.zip › Figure 1/1H/western FLAG.tif]

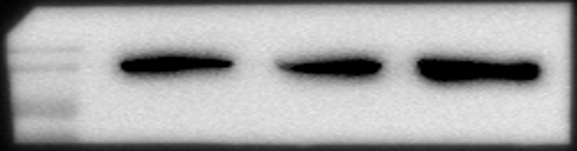

Supplement: Supplementary file 2 — Source data Fig. 1 [file 44319_2024_210_MOESM2_ESM.zip › Figure 1/1H/western SMC3.tif]

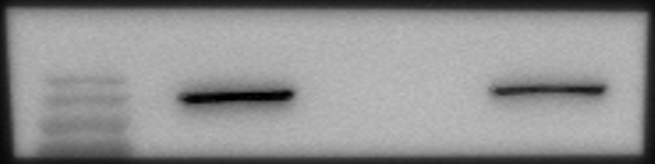

Supplement: Supplementary file 2 — Source data Fig. 1 [file 44319_2024_210_MOESM2_ESM.zip › Figure 1/1I/western AcSMC3.tif]

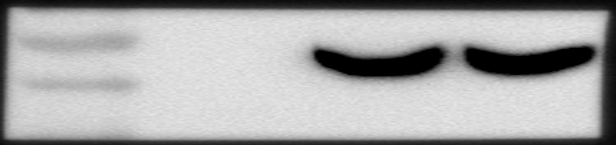

Supplement: Supplementary file 2 — Source data Fig. 1 [file 44319_2024_210_MOESM2_ESM.zip › Figure 1/1I/western HIS.tif]

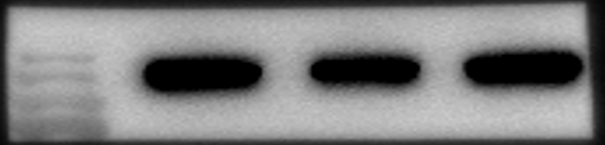

Supplement: Supplementary file 2 — Source data Fig. 1 [file 44319_2024_210_MOESM2_ESM.zip › Figure 1/1I/western SMC3.tif]

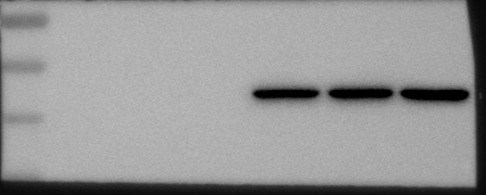

Supplement: Supplementary file 3 — Source data Fig. 2 [file 44319_2024_210_MOESM3_ESM.zip › Figure 2/2A/western IP-FLAG FLAG INPUT.tif]

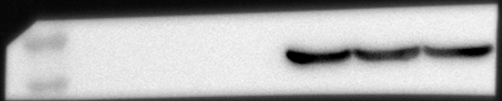

Supplement: Supplementary file 3 — Source data Fig. 2 [file 44319_2024_210_MOESM3_ESM.zip › Figure 2/2A/western IP-FLAG FLAG.tif]

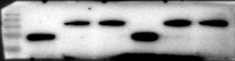

Supplement: Supplementary file 3 — Source data Fig. 2 [file 44319_2024_210_MOESM3_ESM.zip › Figure 2/2A/western IP-FLAG HA INPUT.tif]

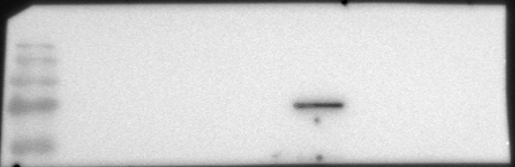

Supplement: Supplementary file 3 — Source data Fig. 2 [file 44319_2024_210_MOESM3_ESM.zip › Figure 2/2A/western IP-FLAG HA.tif]

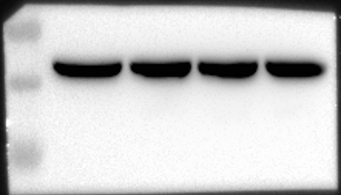

Supplement: Supplementary file 3 — Source data Fig. 2 [file 44319_2024_210_MOESM3_ESM.zip › Figure 2/2A/western IP-HA FLAG INPUT.tif]

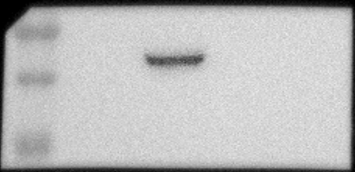

Supplement: Supplementary file 3 — Source data Fig. 2 [file 44319_2024_210_MOESM3_ESM.zip › Figure 2/2A/western IP-HA FLAG.tif]

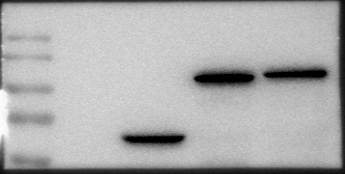

Supplement: Supplementary file 3 — Source data Fig. 2 [file 44319_2024_210_MOESM3_ESM.zip › Figure 2/2A/western IP-HA HA INPUT.tif]

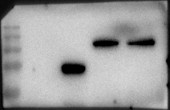

Supplement: Supplementary file 3 — Source data Fig. 2 [file 44319_2024_210_MOESM3_ESM.zip › Figure 2/2A/western IP-HA HA.tif]

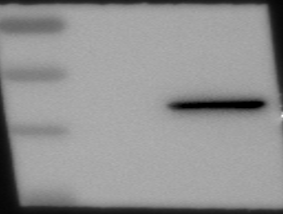

Supplement: Supplementary file 3 — Source data Fig. 2 [file 44319_2024_210_MOESM3_ESM.zip › Figure 2/2B/western IP-FALG FLAG INPUT.tif]

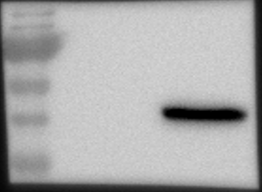

Supplement: Supplementary file 3 — Source data Fig. 2 [file 44319_2024_210_MOESM3_ESM.zip › Figure 2/2B/western IP-FALG FLAG.tif]

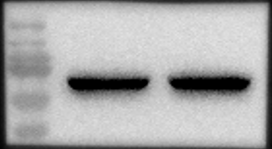

Supplement: Supplementary file 3 — Source data Fig. 2 [file 44319_2024_210_MOESM3_ESM.zip › Figure 2/2B/western IP-FALG Tip60 INPUT.tif]

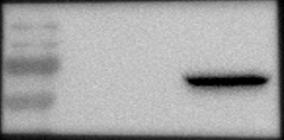

Supplement: Supplementary file 3 — Source data Fig. 2 [file 44319_2024_210_MOESM3_ESM.zip › Figure 2/2B/western IP-FALG Tip60.tif]

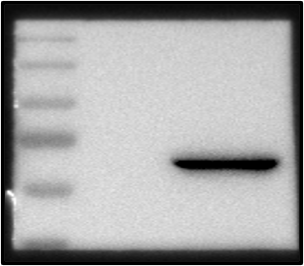

Supplement: Supplementary file 3 — Source data Fig. 2 [file 44319_2024_210_MOESM3_ESM.zip › Figure 2/2B/western IP-HA HA INPUT.tif]

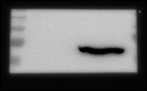

Supplement: Supplementary file 3 — Source data Fig. 2 [file 44319_2024_210_MOESM3_ESM.zip › Figure 2/2B/western IP-HA HA.tif]

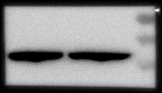

Supplement: Supplementary file 3 — Source data Fig. 2 [file 44319_2024_210_MOESM3_ESM.zip › Figure 2/2B/western IP-HA HDAC8 INPUT.tif]

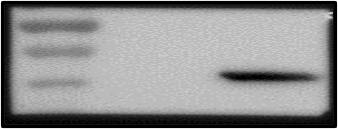

Supplement: Supplementary file 3 — Source data Fig. 2 [file 44319_2024_210_MOESM3_ESM.zip › Figure 2/2B/western IP-HA HDAC8.tif]

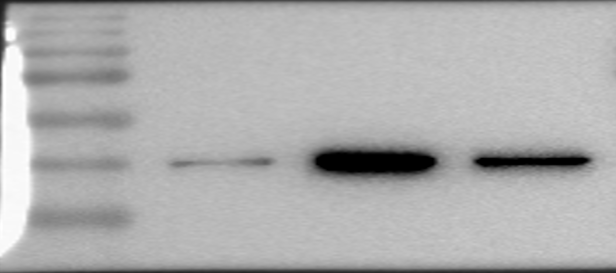

Supplement: Supplementary file 3 — Source data Fig. 2 [file 44319_2024_210_MOESM3_ESM.zip › Figure 2/2C/western AcK.tif]

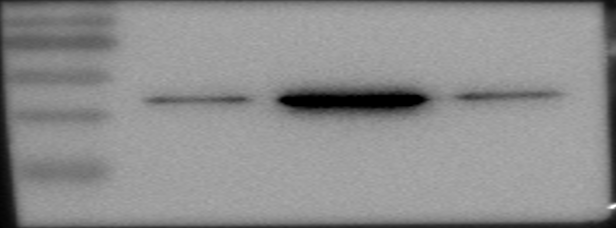

Supplement: Supplementary file 3 — Source data Fig. 2 [file 44319_2024_210_MOESM3_ESM.zip › Figure 2/2C/western AcK202.tif]

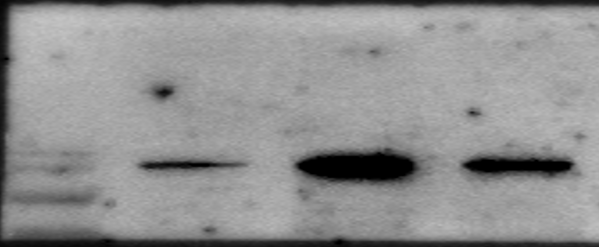

Supplement: Supplementary file 3 — Source data Fig. 2 [file 44319_2024_210_MOESM3_ESM.zip › Figure 2/2C/western AcSMC3.tif]

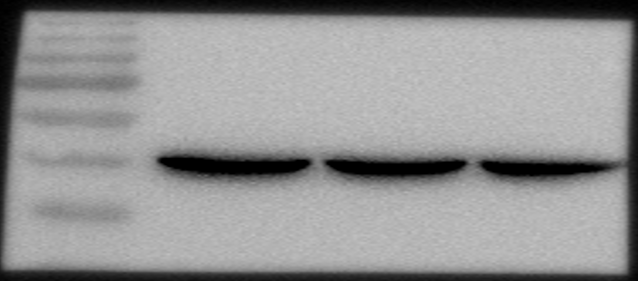

Supplement: Supplementary file 3 — Source data Fig. 2 [file 44319_2024_210_MOESM3_ESM.zip › Figure 2/2C/western Actin.tif]

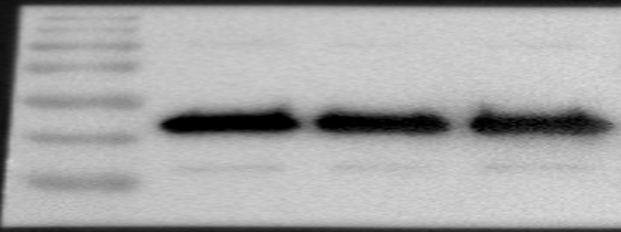

Supplement: Supplementary file 3 — Source data Fig. 2 [file 44319_2024_210_MOESM3_ESM.zip › Figure 2/2C/western FLAG.tif]

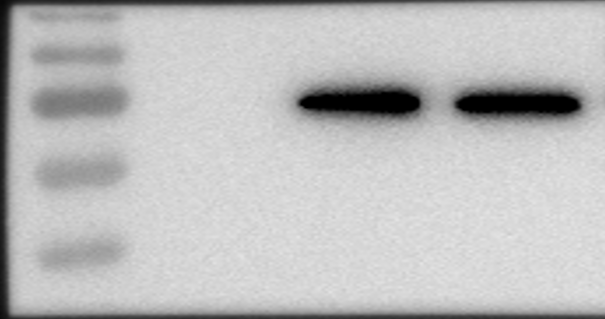

Supplement: Supplementary file 3 — Source data Fig. 2 [file 44319_2024_210_MOESM3_ESM.zip › Figure 2/2C/western HA.tif]

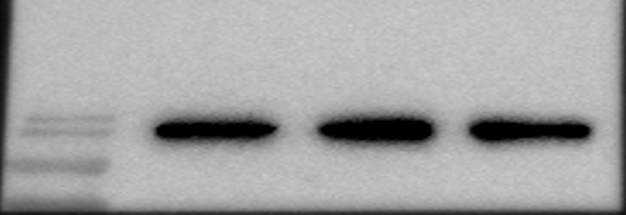

Supplement: Supplementary file 3 — Source data Fig. 2 [file 44319_2024_210_MOESM3_ESM.zip › Figure 2/2C/western SMC3.tif]

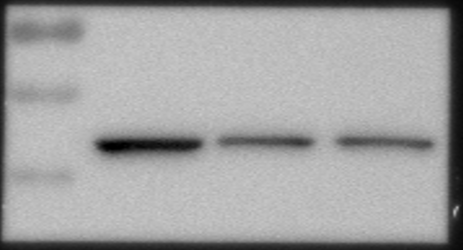

Supplement: Supplementary file 3 — Source data Fig. 2 [file 44319_2024_210_MOESM3_ESM.zip › Figure 2/2D/western AcK.tif]

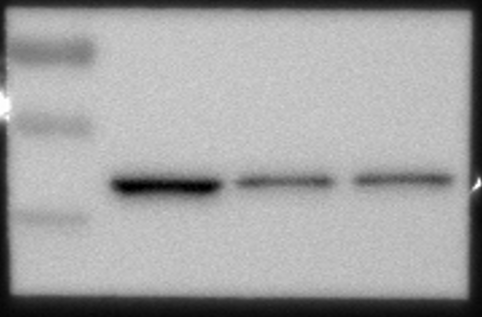

Supplement: Supplementary file 3 — Source data Fig. 2 [file 44319_2024_210_MOESM3_ESM.zip › Figure 2/2D/western AcK202.tif]

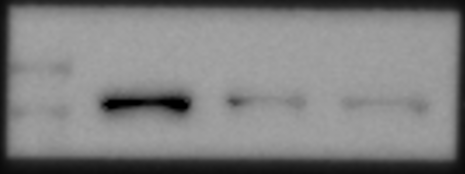

Supplement: Supplementary file 3 — Source data Fig. 2 [file 44319_2024_210_MOESM3_ESM.zip › Figure 2/2D/western AcSMC3.tif]

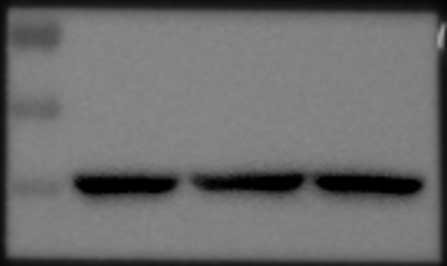

Supplement: Supplementary file 3 — Source data Fig. 2 [file 44319_2024_210_MOESM3_ESM.zip › Figure 2/2D/western Actin.tif]

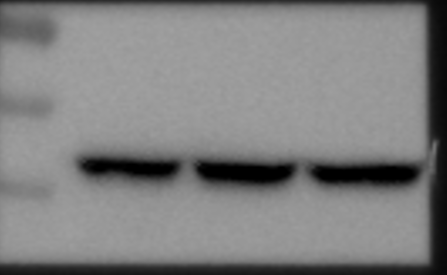

Supplement: Supplementary file 3 — Source data Fig. 2 [file 44319_2024_210_MOESM3_ESM.zip › Figure 2/2D/western HDAC8.tif]

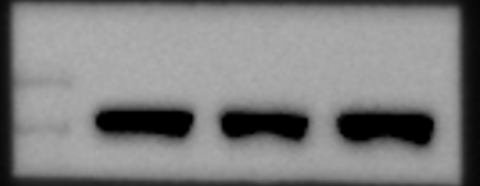

Supplement: Supplementary file 3 — Source data Fig. 2 [file 44319_2024_210_MOESM3_ESM.zip › Figure 2/2D/western SMC3.tif]

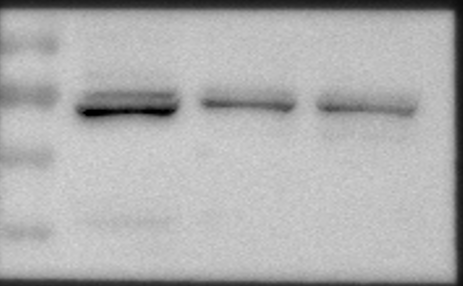

Supplement: Supplementary file 3 — Source data Fig. 2 [file 44319_2024_210_MOESM3_ESM.zip › Figure 2/2D/western Tip60.tif]

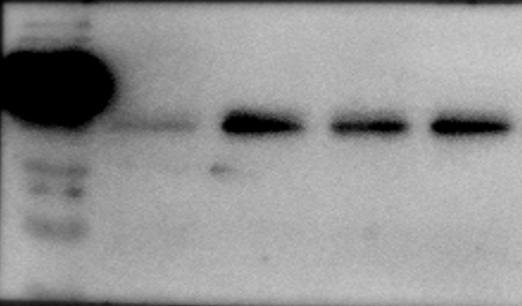

Supplement: Supplementary file 3 — Source data Fig. 2 [file 44319_2024_210_MOESM3_ESM.zip › Figure 2/2F/western AcK.tif]

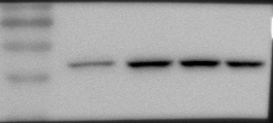

Supplement: Supplementary file 3 — Source data Fig. 2 [file 44319_2024_210_MOESM3_ESM.zip › Figure 2/2F/western AcK202.tif]

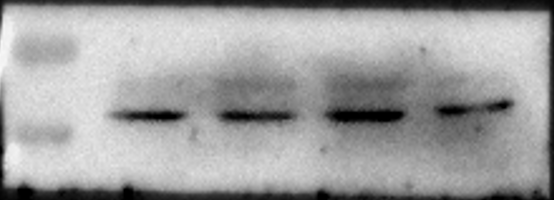

Supplement: Supplementary file 3 — Source data Fig. 2 [file 44319_2024_210_MOESM3_ESM.zip › Figure 2/2F/western Actin.tif]

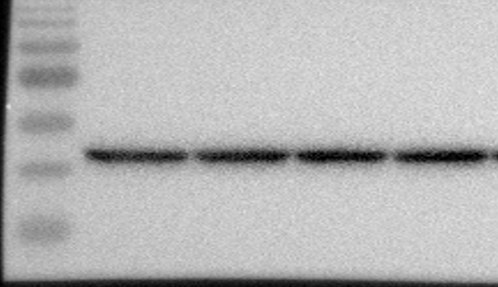

Supplement: Supplementary file 3 — Source data Fig. 2 [file 44319_2024_210_MOESM3_ESM.zip › Figure 2/2F/western FLAG.tif]

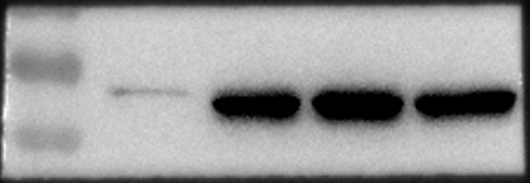

Supplement: Supplementary file 3 — Source data Fig. 2 [file 44319_2024_210_MOESM3_ESM.zip › Figure 2/2F/western Tip60.tif]

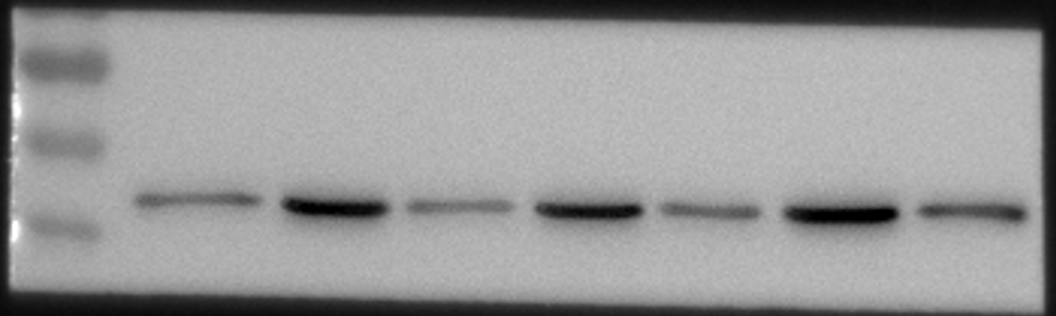

Supplement: Supplementary file 4 — Source data Fig. 3 [file 44319_2024_210_MOESM4_ESM.zip › Figure 3/3A/western AcK202.tif]

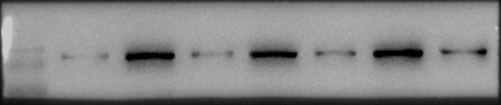

Supplement: Supplementary file 4 — Source data Fig. 3 [file 44319_2024_210_MOESM4_ESM.zip › Figure 3/3A/western AcSMC3.tif]

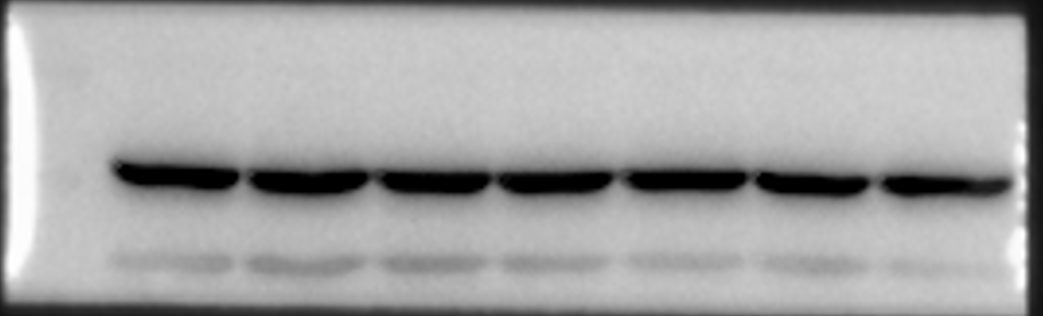

Supplement: Supplementary file 4 — Source data Fig. 3 [file 44319_2024_210_MOESM4_ESM.zip › Figure 3/3A/western Actin.tif]

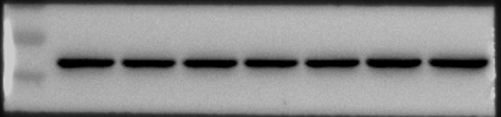

Supplement: Supplementary file 4 — Source data Fig. 3 [file 44319_2024_210_MOESM4_ESM.zip › Figure 3/3A/western HDAC8.tif]

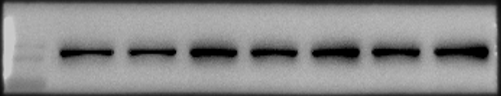

Supplement: Supplementary file 4 — Source data Fig. 3 [file 44319_2024_210_MOESM4_ESM.zip › Figure 3/3A/western SMC3.tif]

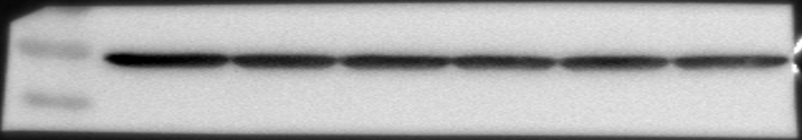

Supplement: Supplementary file 4 — Source data Fig. 3 [file 44319_2024_210_MOESM4_ESM.zip › Figure 3/3E/Tubulin.tif]

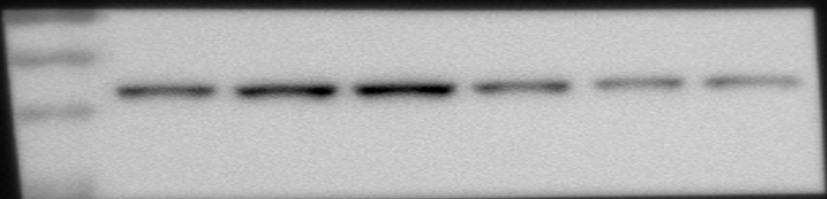

Supplement: Supplementary file 4 — Source data Fig. 3 [file 44319_2024_210_MOESM4_ESM.zip › Figure 3/3E/western AcK202.tif]

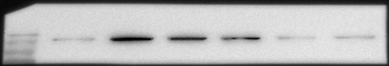

Supplement: Supplementary file 4 — Source data Fig. 3 [file 44319_2024_210_MOESM4_ESM.zip › Figure 3/3E/western AcSMC3.tif]

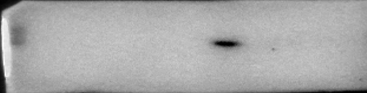

Supplement: Supplementary file 4 — Source data Fig. 3 [file 44319_2024_210_MOESM4_ESM.zip › Figure 3/3E/western H3S10-P.tif]

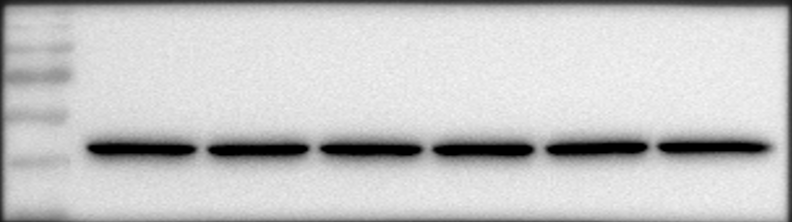

Supplement: Supplementary file 4 — Source data Fig. 3 [file 44319_2024_210_MOESM4_ESM.zip › Figure 3/3E/western HDAC8.tif]

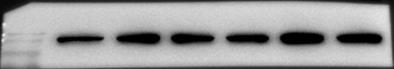

Supplement: Supplementary file 4 — Source data Fig. 3 [file 44319_2024_210_MOESM4_ESM.zip › Figure 3/3E/western SMC3.tif]

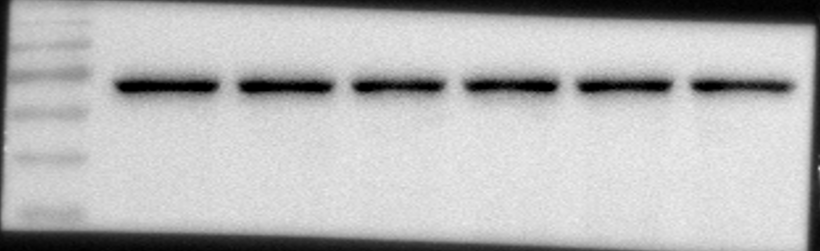

Supplement: Supplementary file 4 — Source data Fig. 3 [file 44319_2024_210_MOESM4_ESM.zip › Figure 3/3E/western Tip60.tif]

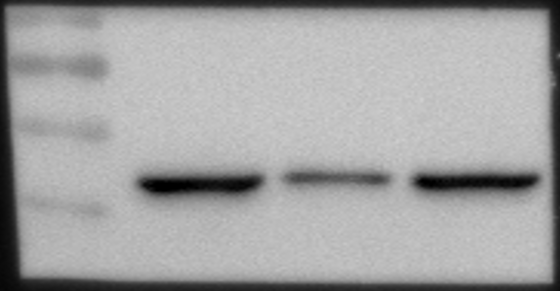

Supplement: Supplementary file 4 — Source data Fig. 3 [file 44319_2024_210_MOESM4_ESM.zip › Figure 3/3F/western AcK202.tif]

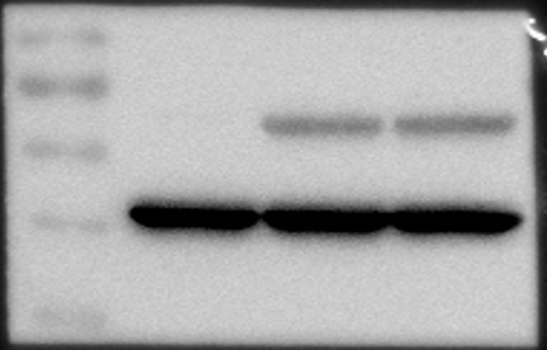

Supplement: Supplementary file 4 — Source data Fig. 3 [file 44319_2024_210_MOESM4_ESM.zip › Figure 3/3F/western HDAC8.tif]

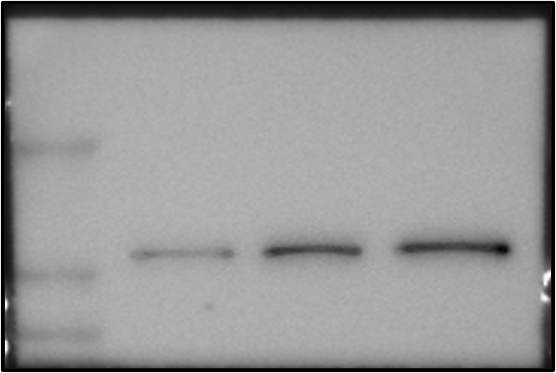

Supplement: Supplementary file 5 — Source data Fig. 4 [file 44319_2024_210_MOESM5_ESM.zip › Figure 4/4B/western AcSMC3.tif]

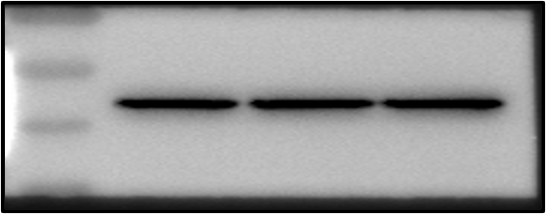

Supplement: Supplementary file 5 — Source data Fig. 4 [file 44319_2024_210_MOESM5_ESM.zip › Figure 4/4B/western HDAC8.tif]

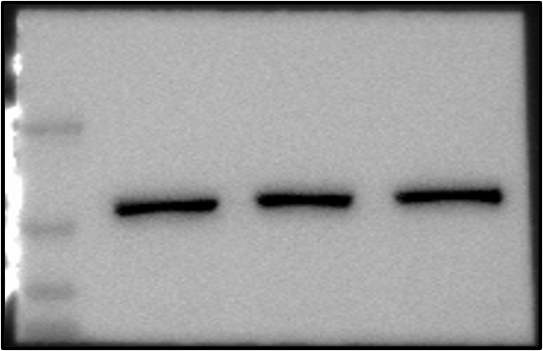

Supplement: Supplementary file 5 — Source data Fig. 4 [file 44319_2024_210_MOESM5_ESM.zip › Figure 4/4B/western SMC3.tif]

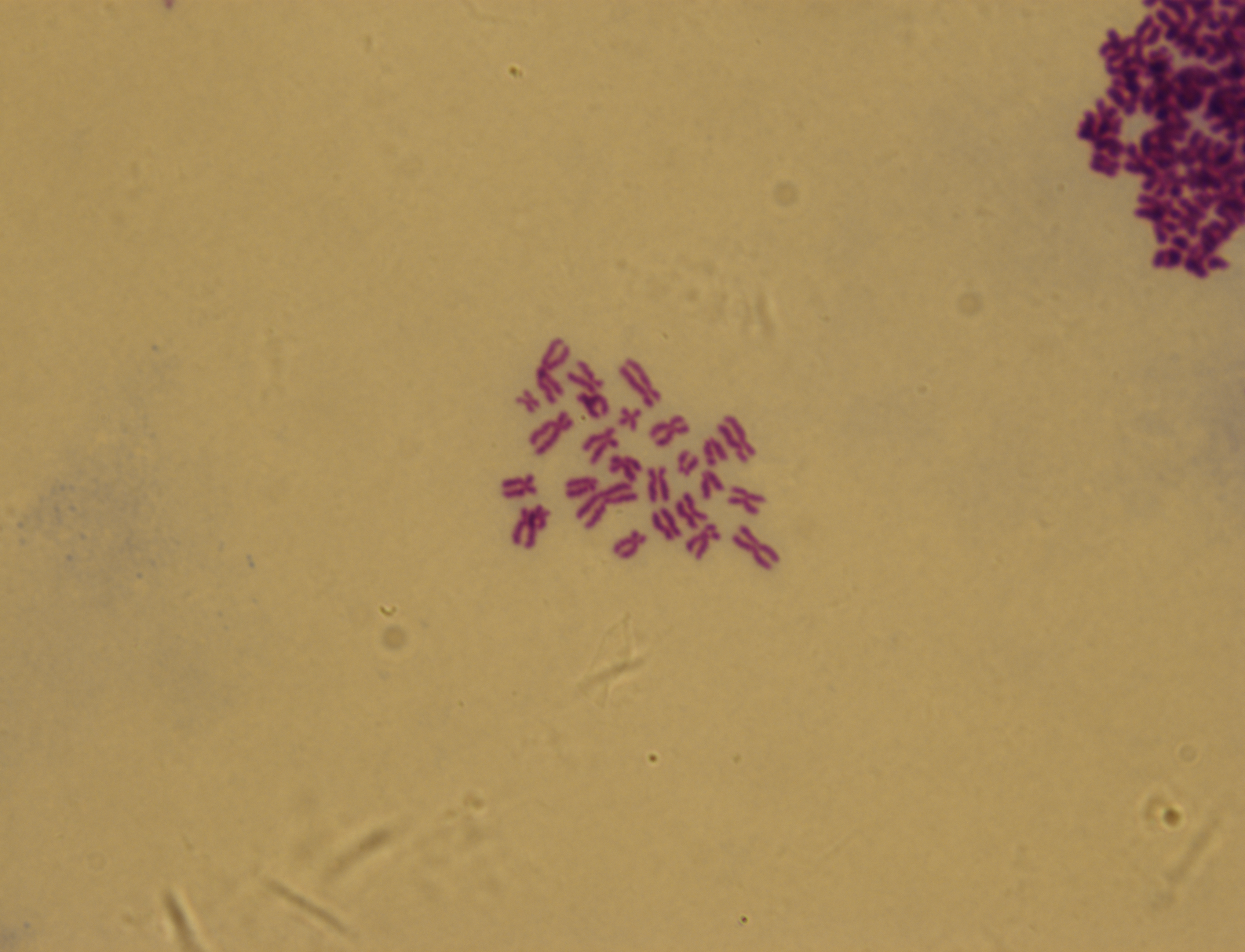

Supplement: Supplementary file 6 — Source data Fig. 5 [file 44319_2024_210_MOESM6_ESM.zip › Figure 5/5E/Mild.tif]

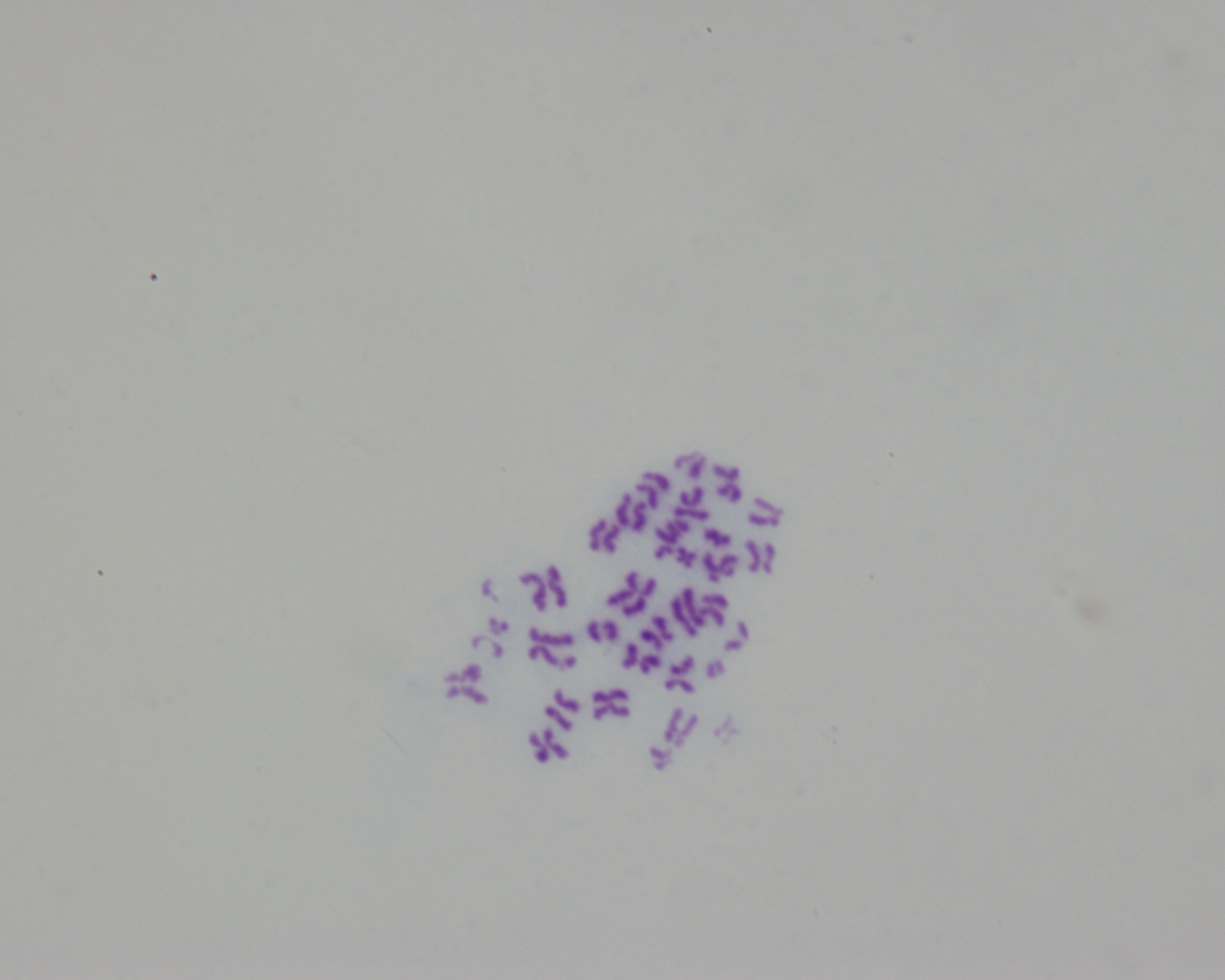

Supplement: Supplementary file 6 — Source data Fig. 5 [file 44319_2024_210_MOESM6_ESM.zip › Figure 5/5E/Moderate.tif]

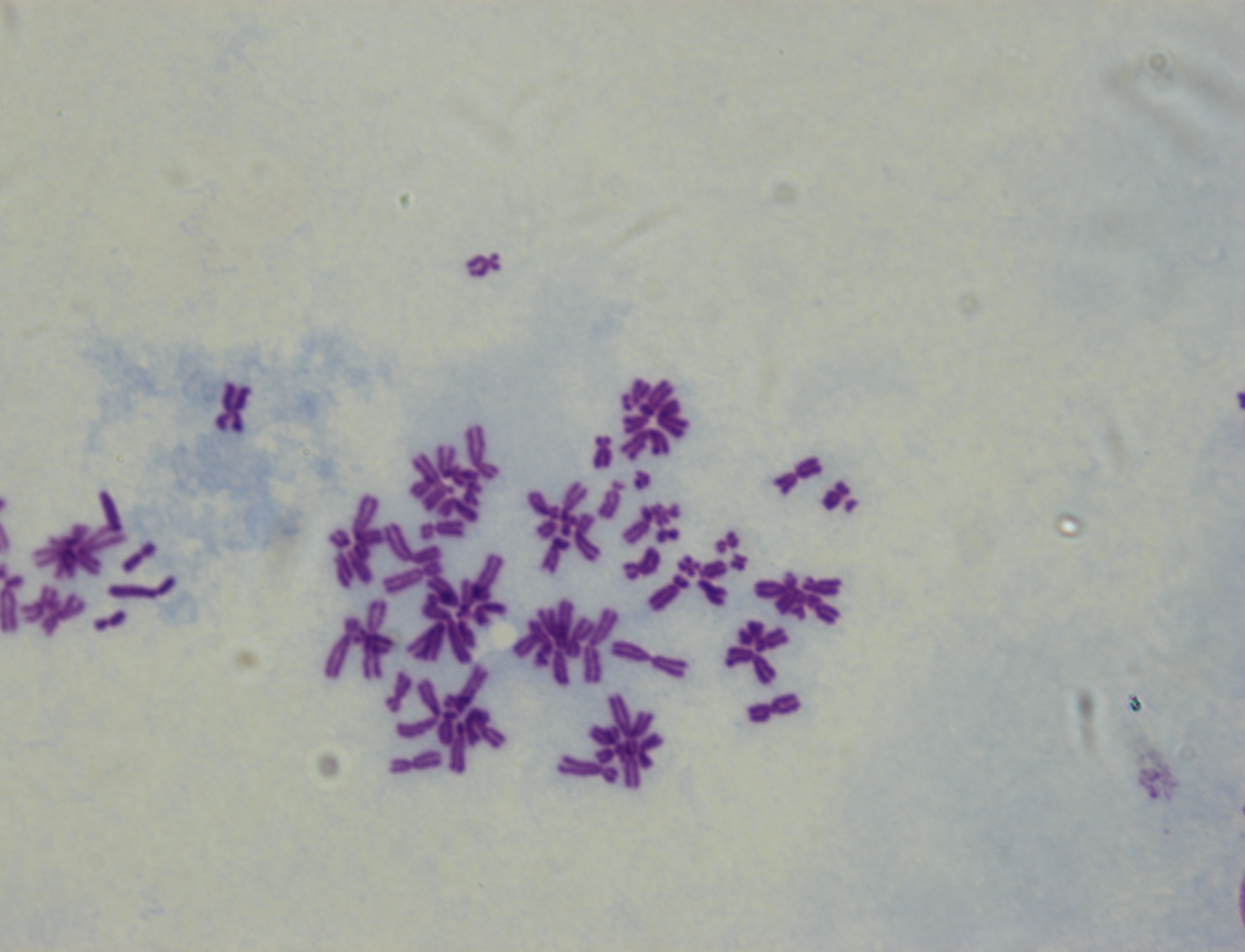

Supplement: Supplementary file 6 — Source data Fig. 5 [file 44319_2024_210_MOESM6_ESM.zip › Figure 5/5E/Normal.tif]

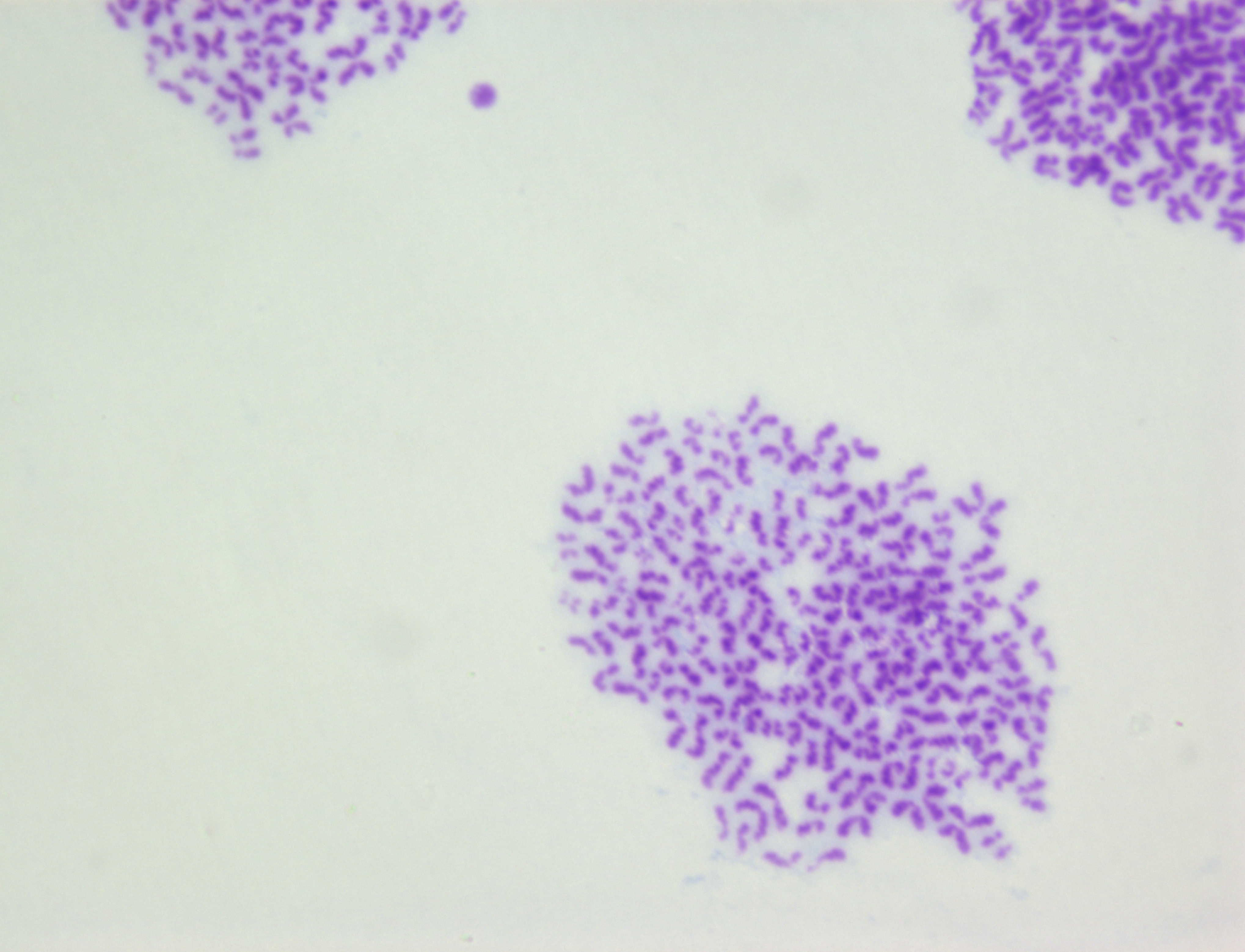

Supplement: Supplementary file 6 — Source data Fig. 5 [file 44319_2024_210_MOESM6_ESM.zip › Figure 5/5E/Severe.tif]
