# Supplementary material for: Reversible acetylation of HDAC8 regulates cell cycle
Source: EMBO Rep. 2024 Jul 23;25(9):13. doi: 10.1038/s44319-024-00210-w (PMC11387496; doi:10.1038/s44319-024-00210-w)
Supplement: Supplementary file 7 — Expanded View Figures [file 44319_2024_210_MOESM7_ESM.pdf]

Expanded View Figures

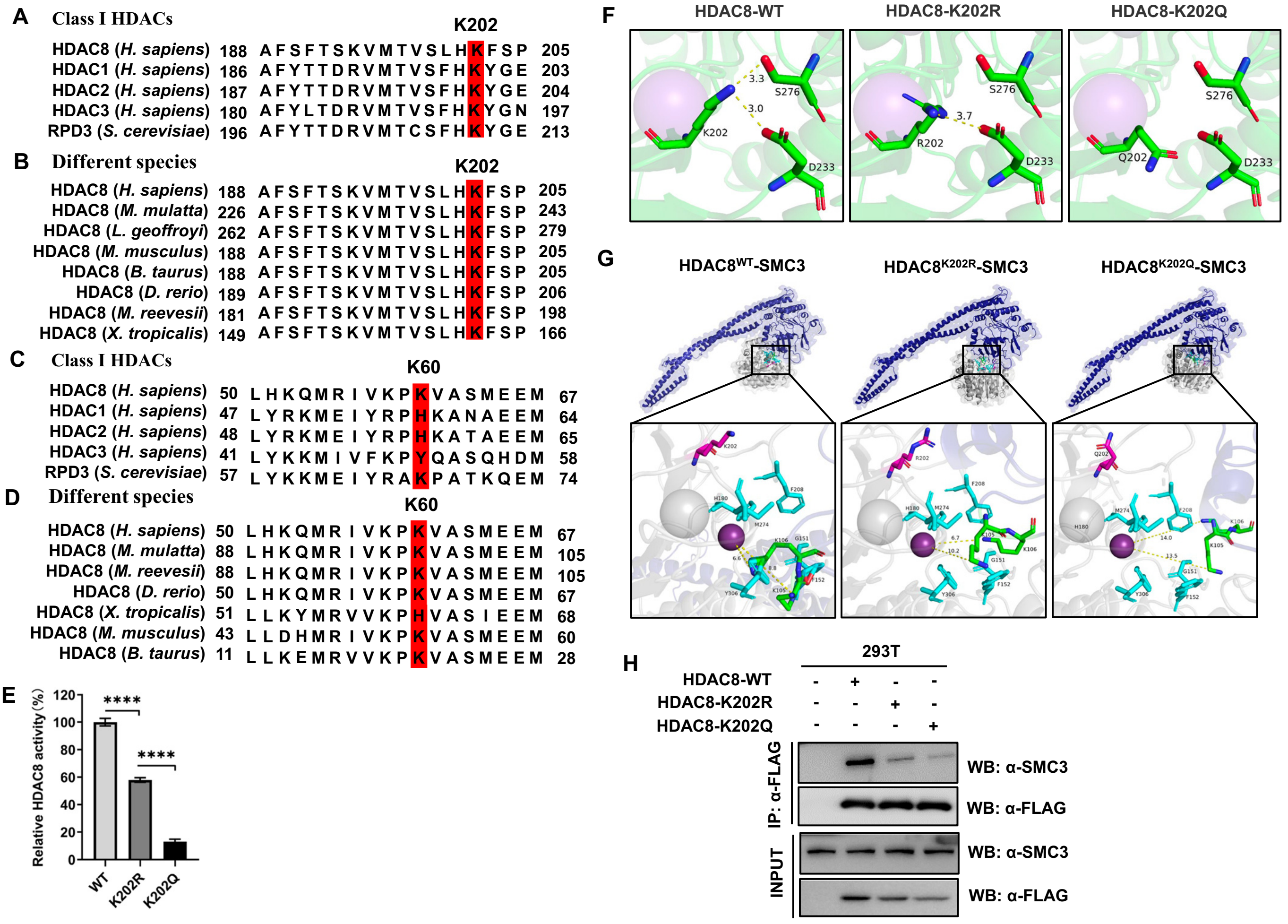

Figure EV1. K202 acetylation disrupts HDAC8 structure and inhibits its activity.

(**A**, **B**) K202 in HDAC8 is evolutionarily conserved. The sequences surrounding K202 in HDAC8 from different class I HDACs (**A**) and species (**B**) were aligned. (**C**, **D**) K60 in HDAC8 is not highly conserved. The sequences surrounding K60 in HDAC8 from different class I HDACs (**C**) and species (**D**) were aligned. (**E**) HDAC8-K202R and K202Q mutations lead to distinct reductions in deacetylase activity compared to WT protein. WT, K202R, and K202Q mutation of HDAC8 proteins were recombinant expressed E. coli, purified using nickel affinity chromatography, and then performed activity assay. Results were presented as the mean ± SD ($n = 3$, technical replicates). Differences between groups were compared using unpaired two-tailed Student's t tests. ****$P < 0.001$. (**F**) HDAC8 K202Q mutation may disrupt the hydrogen bond network involving D233-K202-S276. The crystal structure of HDAC8-WT was obtained from previously published data (PDB accession code 1W22). The K202Q and K202R mutant structures of HDAC8 were predicted using the mutagenesis function of PyMOL based on the HDAC8-WT crystal structure. Dotted yellow lines indicated hydrogen bonds. In HDAC8-WT, robust hydrogen bond interactions were observed between K202-D233 (3.0 Å) and K202-S276 (3.3 Å). Conversely, in HDAC8-K202R, a hydrogen bond interaction was observed only between R202 and D233 (3.7 Å). In HDAC8 K202Q, Q202 failed to form hydrogen bonds with either D233 or S276. (**G**) Crystal structure of HDAC8 (WT, K202R, K202Q) -SMC3 complex predicted by ZDOCK 3.0.2 based on the crystal structure of HDAC8 (PDB accession code 1W22) and SMC3 (PDB accession code 7W1M). Compared to HDAC8-WT, the catalytic binding pockets of HDAC8-K202R and HDAC8 K202Q were increasingly distant from the acetylation sites K105 and K106 of SMC3, respectively. Overall structure of complex in PyMOL, with HDAC8 (WT, K202Q, K202R) and SMC3 shown as surfaces and cartoons. The key site (K202, Q202, R202), pocket walls (G151, F152, H180, F208, M274, Y306) of HDAC8 and the acetylation sites (K105, K106) of SMC3 in the focused panel were shown as sticks and colored in magenta, cyan and green respectively, Zinc ion was shown as a sphere colored in violet and the distance between Zinc ion and K105, K106 was shown. SMC3 was colored in density and HDAC8 (WT, K202Q, K202R) was colored in gray70. (**H**) Representative immunoblotting of 3 independent experiments shows that HDAC8 K202Q mutation causes diminished binding to the substrate SMC3. 293T cells were transfected with an empty vector, Flag-HDAC8-WT, Flag-HDAC8-K202R or Flag-HDAC8 K202Q. Whole-cell lysates were subjected to immunoprecipitation with anti-Flag beads, followed by immunoblot analysis to detect the precipitated proteins.

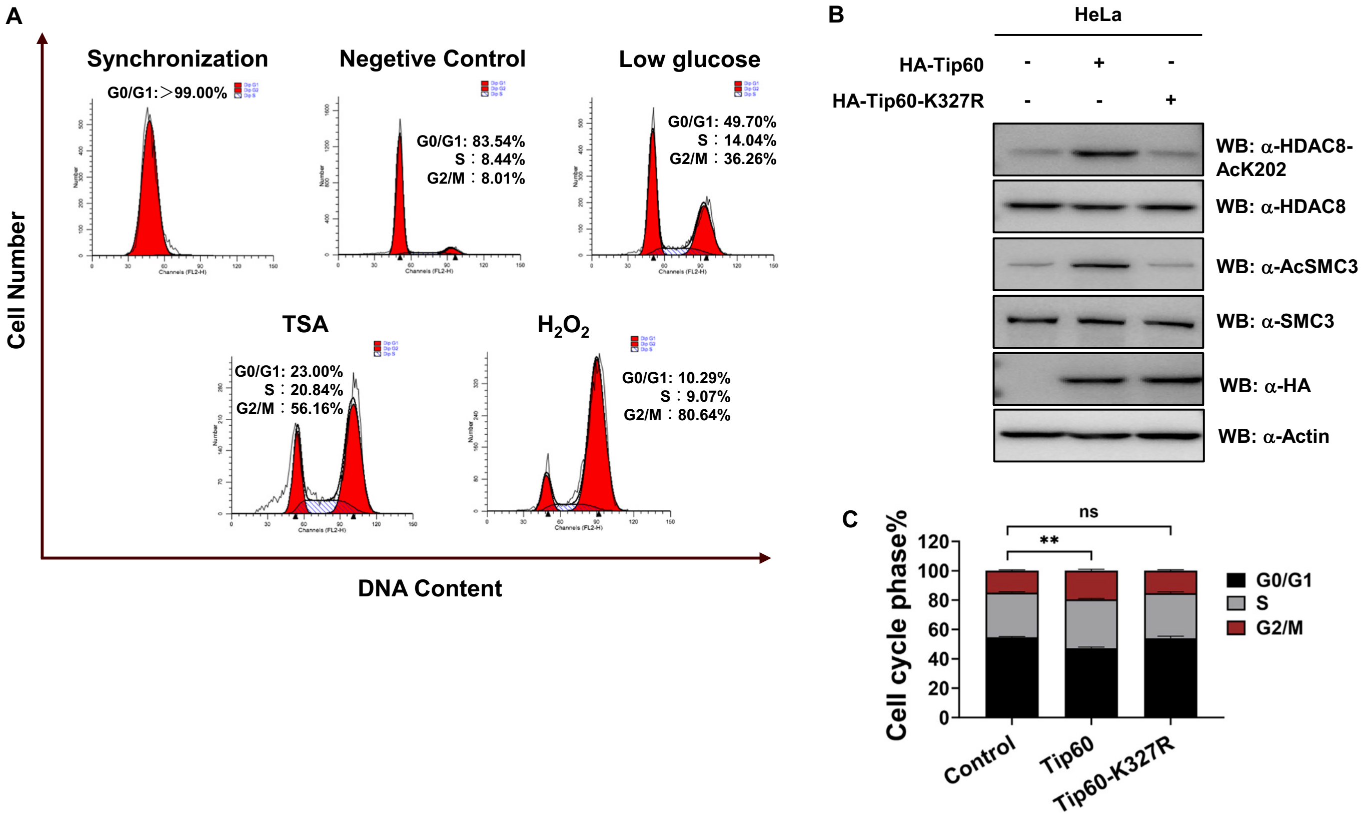

Figure EV2. Overexpression of Tip60 causes elevated HDAC8 acetylation and cell cycle arrest.

(**A**) Representative cell cycle distribution of HeLa cells synchronized in S-phase and treated with TSA, H_2O_2, glucose-free medium, or standard DMEM medium. Cells were initially synchronized in the early S-phase using a double-thymidine arrest and then exposed to TSA (500 nM), H_2O_2 (400 nM), glucose-free medium, or standard DMEM medium (as control) for 15 h. Flow cytometry analysis was performed with PI staining to detect cell cycle distribution. (**B**, **C**) Overexpression of Tip60 causes elevated acetylation levels of HDAC8 and SMC3 with concomitant G2/M phase arrest. HeLa cells were transfected with an empty vector, HA-Tip60 or HA-Tip60-K327R. Cells were harvested to prepare the whole-cell extracts for western blotting (**B**) or stain with PI for flow cytometry analysis (**C**). Results were presented as the mean ± SD ($n = 3$, biological replicates). Differences between groups were compared using unpaired two-tailed Student's *t* tests. $^{**}P < 0.01$; ns not significant.

© The Author(s)

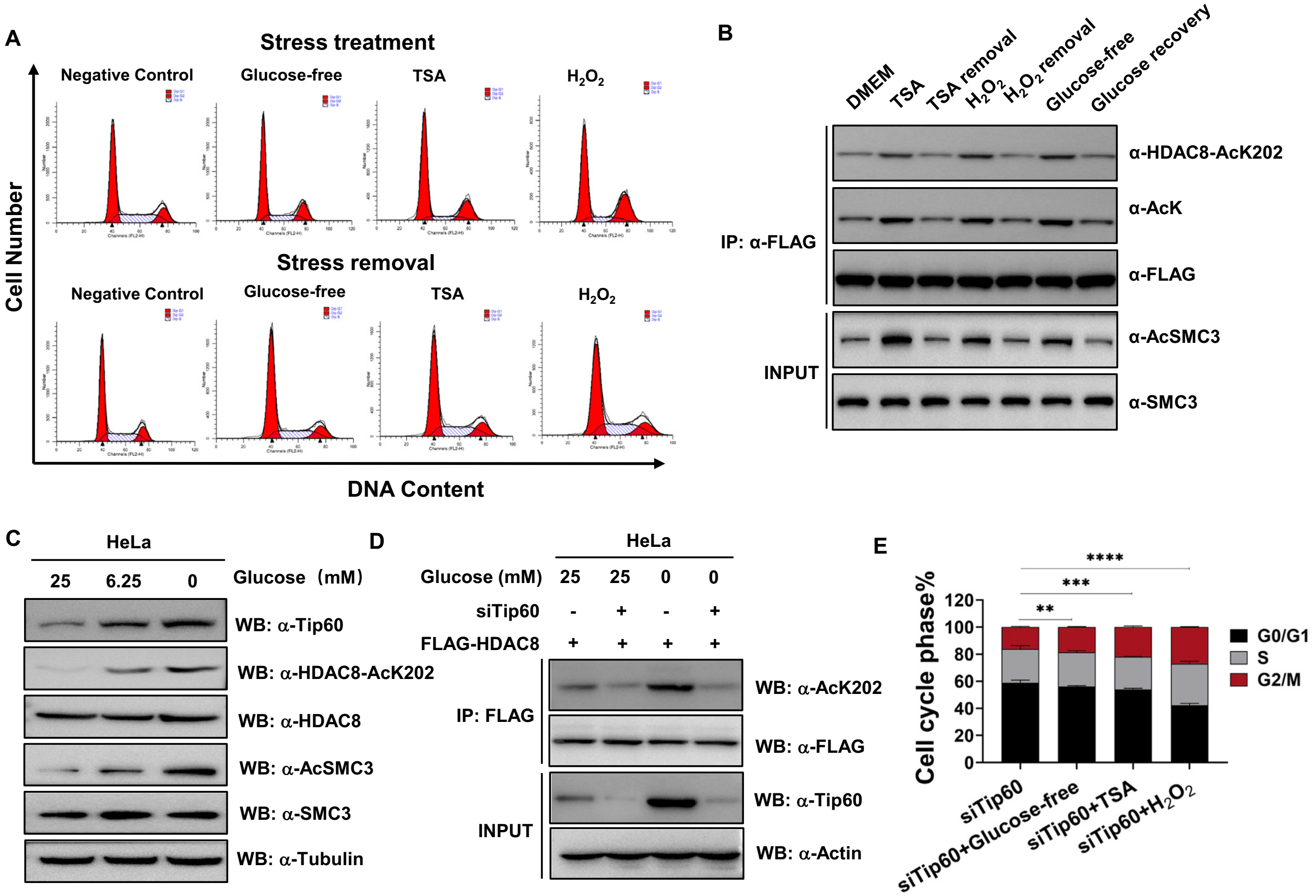

Figure EV3. K202 acetylation of HDAC8 is reversible.

(**A**) Representative cell cycle distribution of HeLa cells after stress treatment and removal. HeLa cells were treated with TSA (500 nM), H_2O_2 (400 nM), glucose-free medium, or standard DMEM medium (as control) for 24 h, and then after removing these stresses, the cells were re-cultured in the standard DMEM medium for about 48 h. Cells were harvested to stain with PI for flow cytometry analysis after stress treatment and removal. (**B**) Representative immunoblotting of 3 independent experiments shows that stress-driven K202 acetylation of ectopically expressed HDAC8 is reversible in HeLa cells. Asynchronous HeLa cells overexpressing Flag-tagged HDAC8 were treated with TSA (500 nM), H_2O_2 (400 nM), glucose-free medium, or standard DMEM medium (as control) for 24 h and then after removing these stresses, the cells were re-cultured in the standard DMEM medium for about 48 h. Whole-cell lysates were subjected to immunoprecipitation with anti-Flag beads, followed by immunoblot analysis to detect the precipitated proteins. (**C**) Representative immunoblotting of 3 independent experiments shows that glucose starvation causes elevated expression of Tip60 and increased acetylation levels of HDAC8 and SMC3 in HeLa cells. Cells were treated with different concentrations of glucose, and then whole-cell extracts were prepared for immunoblot analysis. (**D**) Representative immunoblotting of 3 independent experiments shows that acetylation of HDAC8 is dependent on Tip60 with or without glucose-free stimulation. HeLa cells were transfected with Flag-HDAC8 and Tip60-siRNA, and then cultured with or without glucose condition. Whole-cell lysates were subjected to immunoprecipitation with anti-Flag beads, followed by immunoblot analysis to detect the precipitated proteins. (**E**) Simultaneous knockdown of Tip60 in stress-treated cells does not rescue cell cycle defects. HeLa cells transfected with Tip60-siRNA were treated with TSA (500 nM), H_2O_2 (400 nM), glucose-free medium, or standard DMEM medium (as control) for about 36 h. Cells were harvested for flow cytometry analysis. Results were presented as the mean ± SD ($n = 3$, biological replicates). Differences between groups were compared using unpaired two-tailed Student's t tests. **$P < 0.01$; ***$P < 0.001$; ****$P < 0.0001$.

© The Author(s)

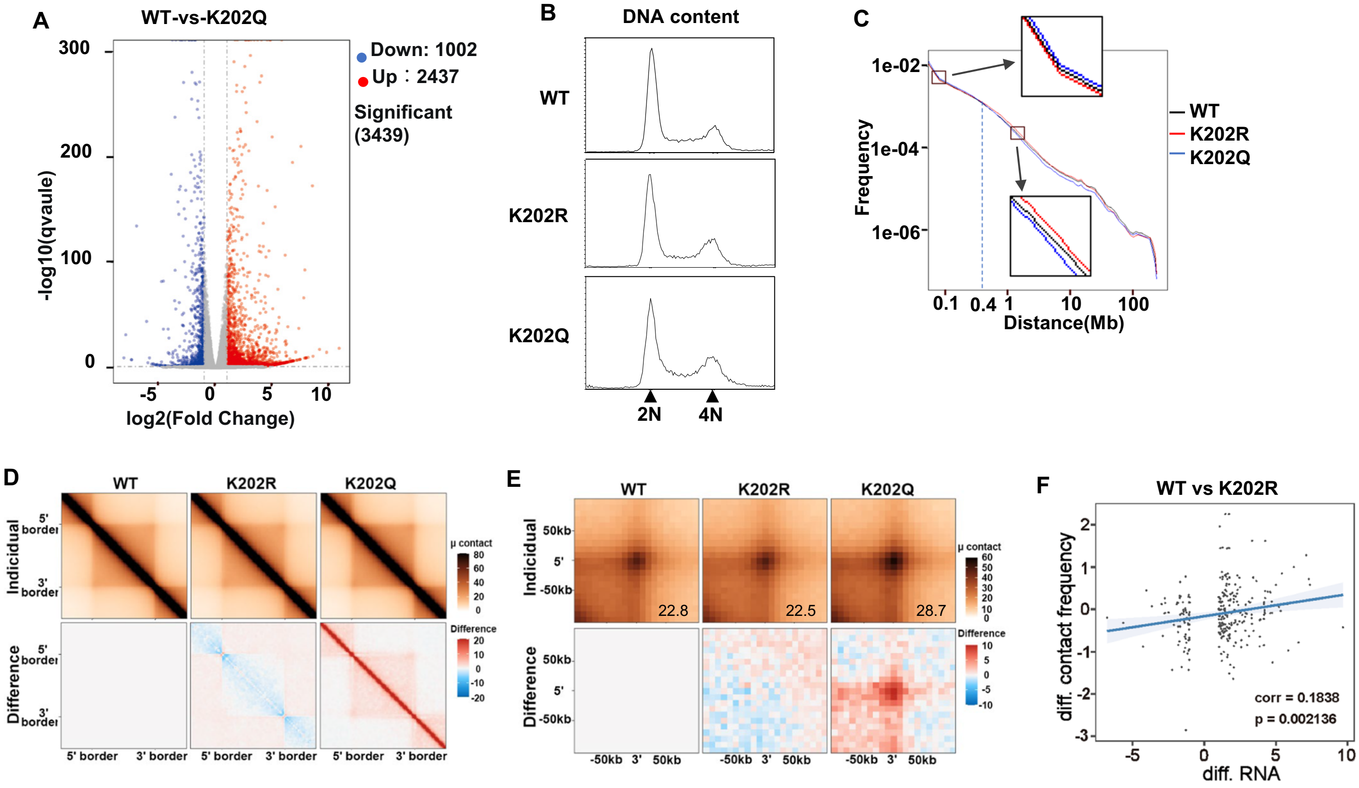

Figure EV4. K202 acetylation promotes chromatin loop formation.

(**A**) Volcano map of differentially expressed genes in the WT and K202Q cells. Significantly differentially expressed genes are shown as a red (up) or blue (down) dot. Non-significantly differentially expressed genes are shown as a gray dot. The horizontal and vertical coordinates represent the change of gene expression multiple and significance, respectively. (**B**) HeLa cells of WT, K202R, and K202Q mutation used for Hi-C experiments have similar cell cycle distributions as shown by flow cytometry analysis. (**C–E**) Another independent biological replicate of the Hi-C experiment for WT, K202R and K202Q HeLa cells, analyzed as in (**D–F**), respectively. Valid contacts were normalized to 56 million among the three samples. (**F**) Scatterplot for the difference of the number of identified significant chromatin interactions (*y* axis) and the difference in gene expression (*x* axis, merged by log2(FPKM + 1)) between WT and K202R cells. The blue solid line represents the fitted linear line, suggesting that the change of significant chromatin interactions positively correlates with the change in gene expression.

 © The Author(s)

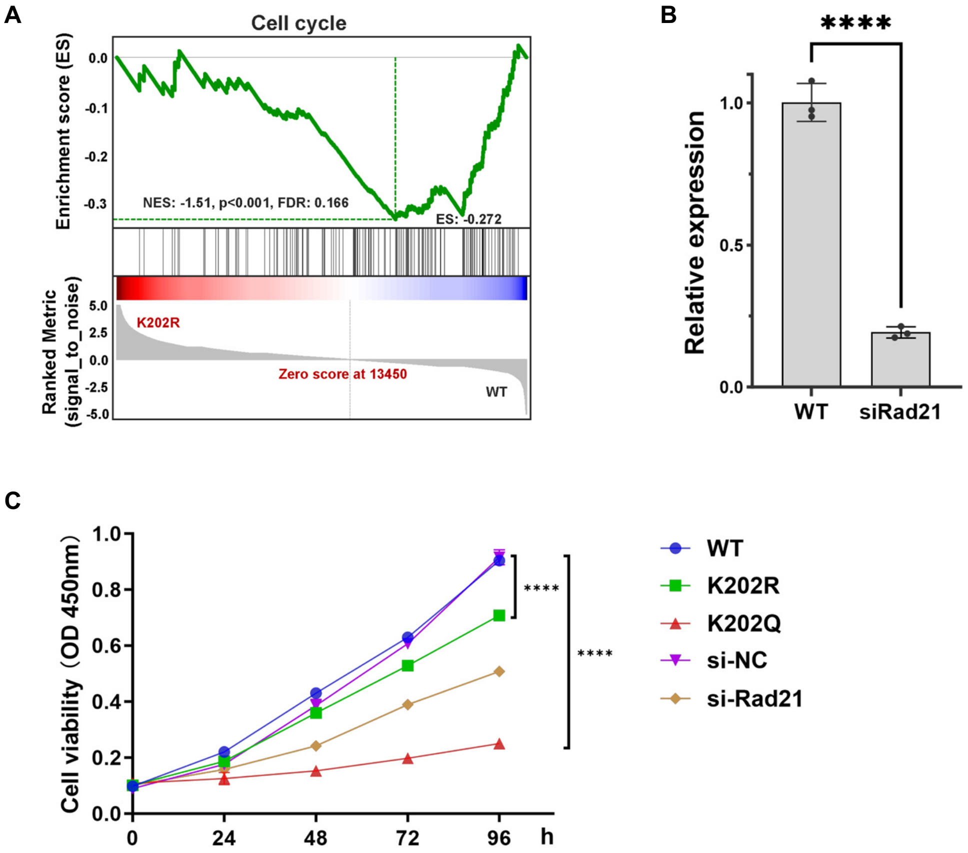

Figure EV5. K202 acetylation of HDAC8 inhibits cell proliferation.

(**A**) Gene set enrichment analysis (GSEA) result of RNA-seq data in the HeLa cells of K202R mutation with WT. The cell cycle signaling pathway is enriched with an FDR of 0.166. (**B**) Knockdown efficiency in Rad21 expression by siRNA was determined by RT-qPCR. Rad21 relative expression levels were calculated using the ΔΔCt method and then normalized to the endogenous reference gene GAPDH. Results are presented as mean ± SD ($n = 3$, biological replicates). ****$P < 0.0001$. (**C**) HDAC8 K202Q mutation inhibits cell proliferation. Cell proliferation of WT, K202R, and K202Q cells was detected by CCK-8 assay, while HeLa cells transfected with siRNA for Rad21 knockdown were used as positive control. Results were presented as the mean ± SD ($n = 3$, technical replicates). Differences between groups were compared using unpaired two-tailed Student's t tests. ****$P < 0.0001$.

© The Author(s)
